# Ten-Year Follow-Up of Collision Tumors Composed of Craniopharyngioma and Pituitary Adenoma: A Case Report and Literature Review

**DOI:** 10.1155/2019/8080163

**Published:** 2019-07-17

**Authors:** Takeshi Miyazaki, Kentaro Kowari, Hirotake Eda, Mizuki Kambara, Riruke Maruyama, Yasuhiko Akiyama

**Affiliations:** ^1^Department of Neurosurgery, Shimane University Faculty of Medicine, 89-1 Enya, Izumo, Shimane 693-8501, Japan; ^2^Department of Neurosurgery, Sakurakai Hospital, 5-2610-1 Handa, Sayama, Osaka 589-0011, Japan; ^3^Department of Organ Pathology, Shimane University Faculty of Medicine, 89-1 Enya, Izumo, Shimane 693-8501, Japan

## Abstract

Although craniopharyngioma (CP) and pituitary adenoma (PA) are common tumors of the parasellar lesions, the coexistence of CP and PA is very rare. A 48-year-old male visited our hospital because of consciousness disturbance. The neuroimaging revealed a sellar tumor contact with a massive suprasellar cyst including calcification. Preoperative diagnosis was CP, and the patient underwent craniotomy to resolve the suprasellar mass effect. The histological examination disclosed adamantinomatous CP, and subsequently a transsphenoidal approach was chosen for the residual intrasellar tumor. Against expectations, the histological diagnosis was not CP but PA. The patient underwent gamma knife surgery for the residual tumor, and the postoperative course was good. After a 10-year follow-up, both lesions were still completely controlled. If we had suspected and diagnosed the tumor involved as not only CP but also PA at the first operation, the second operation could have been avoided because we would have chosen gamma knife surgery for the residual tumor. We should draw attention to this rare situation for differential diagnosis of parasellar tumor to avoid unnecessary surgery and to decide the best strategy for treatment. In addition, the biological behavior of collision tumors composed of CP and PA is probably the same as solitary CP or PA based on a long-term follow-up of our case.

## 1. Introduction

Although craniopharyngioma (CP) and pituitary adenoma (PA) are common tumors of the sellar or suprasellar lesions, the coexistence of CP and PA is very rare. The lack of attention on this rare condition may occasionally bring unnecessary surgery to the patients. To the best of our knowledge, there have been 14 reports involving a CP and a concomitant PA. Herein, we present 15 cases of collision tumors composed of CP and PA with a 10-year follow-up. The clinical features and treatment strategy of these collision tumors are discussed along with a literature review.

## 2. Case Presentation

A 48-year-old male visited our hospital because of remissness and memory disturbance lasting up to several weeks. First, he had been suspected of suffering from sleep apnea syndrome and was psychiatrically hospitalized. Intracranial magnetic resonance images (MRI) for screening revealed an intra- and suprasellar mass in contact with a large cyst, and he was referred to our division.

**Figure 1:**
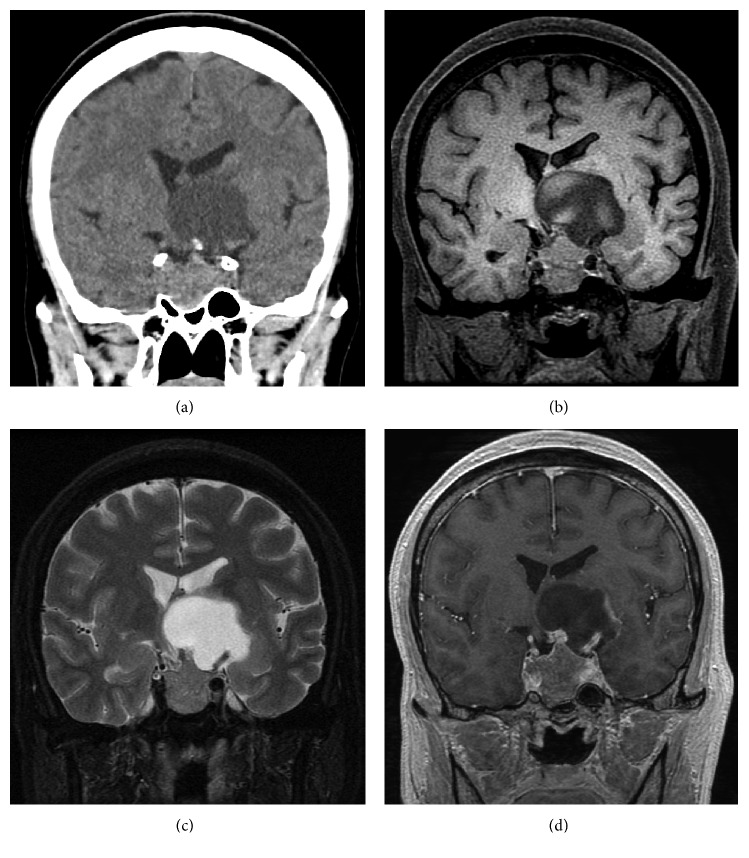
Coronal view of initial CT (a). Initial MRIs: T1-weighted (b), T2-weighted (c), and Gd-enhanced T1-weighted (d) coronal images.

Neurological examination demonstrated mild memory disturbance and right homonymous hemianopsia. A computerized tomography (CT) scan and MRI revealed a massive sellar tumor in contact with a massive suprasellar cyst extending beyond the bilateral cavernous sinus and into the body of the left lateral ventricle. The midline structures (e.g., midbrain and hypothalamus) were severely displaced by the cyst. Part of the cyst wall was calcified on the CT scan (Figure 1(a)). The sellar tumor showed isointensities and slightly high intensities on T1-weighted (Figure 1(b)) and T2-weighted (Figure 1(c)) images, respectively. The suprasellar cyst showed low and high intensities on T1-weighted (Figure 1(b)) and T2-weighted (Figure 1(c)) images, respectively. A gadolinium-diethylenetriaminepentaacetic acid-enhanced T1-weighted image showed the sellar tumor mass as homogenously enhanced and the wall of the suprasellar cyst as slightly enhanced (Figure 1(d)).

Although the baseline values of the pituitary glands were almost within the normal range (growth hormone (GH): 2.2 (normal < 3.0 ng/ml), adrenocorticotropic hormone (ACTH): 43 (normal < 60 pg/ml), thyroid-stimulating hormone (TSH): 4.09 (normal 0.50 to 3.00 *μ*U/ml), luteinizing hormone (LH): 2.6 (normal 1.71 to 8.59 mIU/ml), follicle-stimulating hormone (FSH): 2.8 (normal 1.5 to 12.4 mIU/ml), and prolactin (PRL): 22.0 (normal 3.1 to 20.5 ng/ml)), the pituitary responsiveness was disturbed for LH, FSH, GH, PRL, and cortisol.

**Figure 2:**
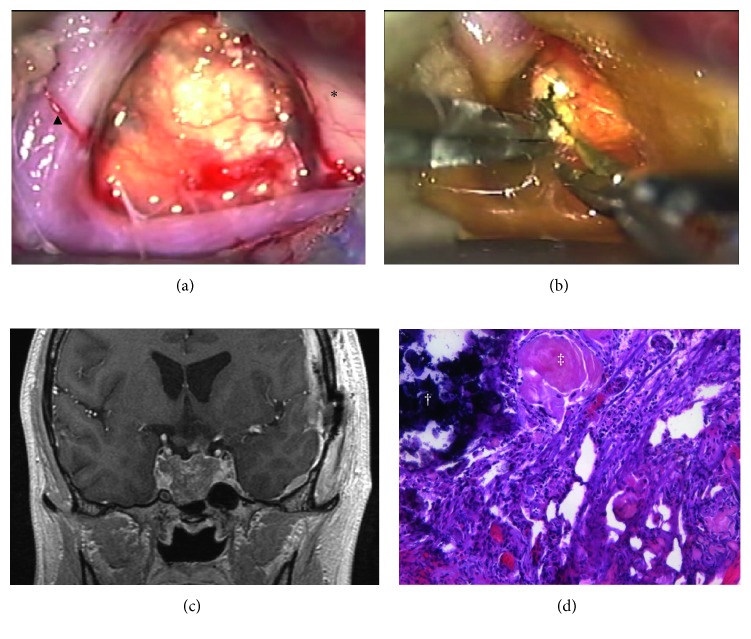
Intraoperative view showing the calcified cyst wall between the left internal carotid artery (▲) and left optic nerve (*∗*) (a). The cyst contents were a motor-oil-like fluid, which indicated a typical craniopharyngioma (b). Postoperative MRI showing reduction of the suprasellar cyst and recovery of the midline structure (c). Photomicrograph of the pathological specimen at the first operation showing features of craniopharyngioma (hematoxylin-eosin staining, magnification ratio 100-fold) (d). The presence of sheets of squamous epithelial cells, calcification (†), and brightly eosinophilic cytoplasm, termed wet keratin (‡), indicates an adamantinomatous type of craniopharyngioma.

Preoperative diagnosis was an intrasellar CP with a large cyst extending toward a suprasellar lesion. However, just after the surgery planning for total resection had been discussed, the patient's level of consciousness became depressed, and right hemiplegia appeared acutely. Therefore, the patient first underwent an emergency operation on the suprasellar tumor and cyst to decrease the intracranial pressure. We chose a left pterional approach because of the cyst's laterality. A left frontoparietal craniotomy and wide open of the left Sylvian fissure exposed the yellowish-white bulging wall of the suprasellar cyst (Figure 2(a)). As extensively as possible, removal of the cyst wall and its calcified nodule was performed. A yellowish semitransparent fluid was aspirated during the excision (Figure 2(b)). Postoperative imaging showed marked cyst shrinkage and decompression of the midbrain and hypothalamus (Figure 2(c)). The origin of the suprasellar tumor was assumed to be the pituitary stalk from the intraoperative findings. The patient's consciousness and right hemiplegia recovered, and memory disturbance and right homonymous hemianopsia also improved postoperatively. Histological examination disclosed adamantinomatous CP as expected (Figure 2(d)).

**Figure 3:**
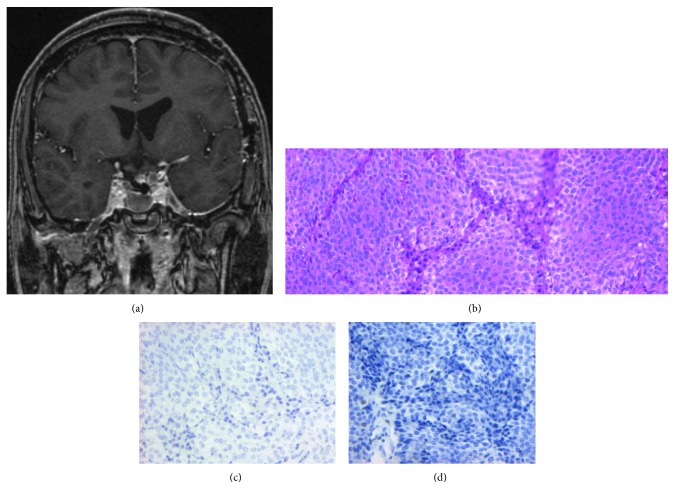
A Gd-enhanced T1-weighted image after transsphenoidal surgery showing reduction of the sellar tumor and enough gain between the sellar tumor and optic chiasma (a). Photomicrograph of the pathological specimen at the second operation showing features of the pituitary adenoma. (b) Hematoxylin-eosin staining, (c) immunostaining for LH, and (d) FSH. The optical magnification ratio of all photomicrographs is 100-fold.

Subsequently, a transsphenoidal approach was chosen as the second operation for the residual tumor. Contrary to our expectations, a rapid histological diagnosis during the second operation was not CP but PA. In consideration of the diagnosis, removal of the intrasellar tumor except for parts of the cavernous sinus invasion was performed (Figure 3(a)). The final histological diagnosis after the second operation was PA (Figure 3(b)). The immunostaining for LH (Figure 3(c)) and FSH (Figure 3(d)) was both negative.

**Figure 4:**
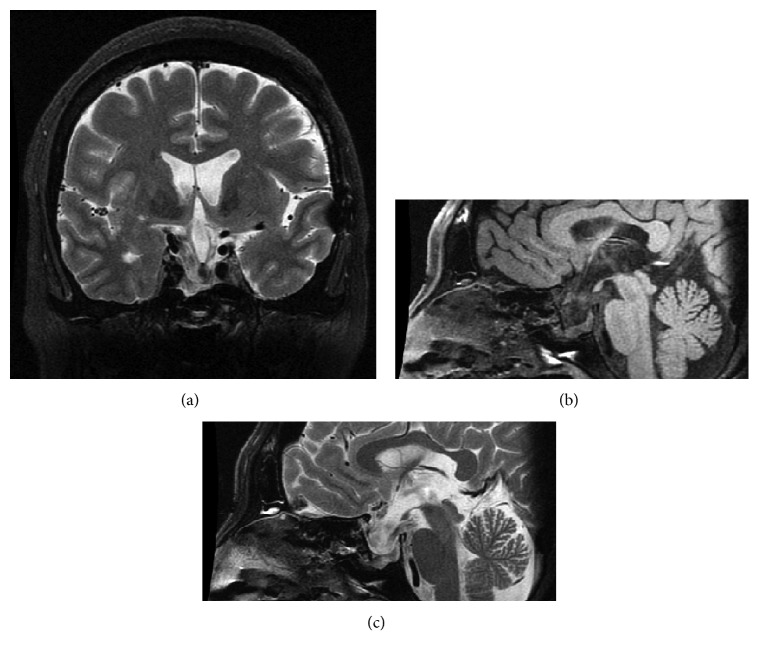
Follow-up MRI of T2-coronal (a), T1-sagittal (b), and T2-sagittal (c) at 10 years after the initial operation showing that the suprasellar cyst and sellar tumor were still completely controlled.

Postoperative course was good. Diabetes insipidus did not occur, and the baseline values of the pituitary gland have been kept at an almost normal range. The patient underwent gamma knife surgery (marginal dose: 10 Gy) to both the residual intra- and suprasellar tumors. After a 10-year follow-up at our outpatient clinic, both lesions were still completely controlled (Figures 4(a)–4(c)).

## 3. Discussion

There have been 14 reports involving a CP and a concomitant PA. Their clinicopathological features are summarized in Table 1. Literature review for the collision tumors composed of CP and PA [1–14]. Based on the fact that both CP and PA are derived from the same origin, the so-called Rathke's pouch, it is no wonder that CP and PA coexist together [15, 16]. Rather, the coexistence of CP and PA might be more frequent, although we are unaware of that situation. As a side note, 8 cases of the past 14 reported cases were cases of prolactinoma. Cusimano et al. considered that destruction of central dopaminergic tuberoinfundibular neurons by CP alters central neurotransmitter regulation of both secretion and division in prolactin-secreting cells and may play a role in the pathogenesis of prolactinoma [7]. However, in reality, CP and PA must coexist incidentally because there are other types of PA, such as gonadotropin-producing or nonfunctioning PA.

As for radiological findings, it is very difficult to detect the coexistence of CP and PA. In fact, all reported 14 cases failed not only to detect but also to suspect the coexistence of CP and PA before the surgery. In particular, in 8 cases for which the tumors were “encased” with each other, it was all but impossible to prove their coexistence until a precise pathological examination was done. On the other hand, there were 7 cases for which the tumors were “separated” and existing side-by-side. In these cases, it might be possible to suspect the coexistence of CP and PA radiologically if we bear in mind the possibility of collision tumor at parasellar lesion.

The prognosis of collision tumors composed of CP and PA had been very poor, particularly before 1990, because patients were forced to undergo multiple surgeries and experienced severe postoperative complications. Since then, a favorable prognosis has been reported. However, Jin et al. reported a case of a patient who was forced to undergo two surgeries because the coexistence of CP was not detected even after the first surgery [14]. They considered that if the coexistence of CP with PA could be established after the first surgery, the second surgery would not be needed. Similarly, if we had suspected the possibility that the tumor involved was not only CP but also PA, we would have tried a biopsy for the deep part of the intrasellar tumor at the first operation. If we had done so, the second operation (transsphenoidal operation) would not have been necessary. This is because the PA had not compressed the optic nerve and was not exerting any adverse effects at that point. So, addition of a little enucleation into the intrasellar tumor could have gained enough distance for stereotactic radiosurgery for PA. The patient could have maybe chosen gamma knife surgery from the beginning because a better control rate of gamma knife to nonfunctioning PA than CP has been reported [17, 18] (in those days, the local control rate for 5 years was almost 90% and 70% for PA and CP, respectively).

In summary, we report a very rare collision tumor composed of a large cystic CP and nonfunctioning PA. It could be suspected that this case was CP concomitant with PA by preoperative MRI retrospectively. We should draw attention to this rare situation for differential diagnosis of a parasellar tumor to avoid unnecessary surgery and to decide the best strategy for treatment. Finally, this is the first case of collision tumors composed of CP and PA with a very long 10-year follow-up period. Based on the clinical course of our case, the best choice of an individual treatment plan for CP or PA is certain to lead to good control for collision tumors composed of CP and PA. The biological behavior of a collision tumor composed of CP and PA is probably the same as solitary CP or PA.

## Figures and Tables

**Table 1:** Literature review for the collision tumors composed of CP and PA.

Case no., author, year	Age, sex	Initial symptom	Initial diagnosis	Hormone	1^st^ treatment	2^nd^ treatment	Pathology of PA	Pathology of CP	Positional relation for CP and PA	Follow-up duration/prognosis after surgery
1, Prabhakar et al., 1971 [1]	29, M	Acromegaly, headache, visual disturbance	PA	GH	Craniotomy	None	GH producing	Adamantinomatous^*∗*^	Separate^*∗*^	4 days/dead due to diabetes insipidus
2, Shishikina et al., 1981 [2]	57, M	Headache, double vision, visual disturbance	CP	PRL (5908 mU/ml)	Craniotomy	Craniotomy	PRL producing	Adamantinomatous^*∗*^	Separate	10 days/dead due to panhypopituitarism
3, Wheatley et al., 1986 [3]	61, M	Headache, visual disturbance, reduced libido	CP	PRL (8180 mU/ml)	Craniotomy	VP shunt	PRL producing	Adamantinomatous^*∗*^	Separate	2 months/dead due to sudden cardiac arrest
4, Dong et al., 1986 [4]	32, F	Acromegaly, amenorrhea, lactation	PA	GH PRL	Transsphenoidal	Craniotomy	GH/PRL producing	Adamantinomatous^*∗*^	Separate^*∗*^	n.d./n.d.
5, Asari et al., 1987 [5]	47, M	Visual disturbance	PA	PRL (360 ng/ml)	Transsphenoidal	Craniotomy (3^rd^ craniotomy)	PRL producing	Adamantinomatous^*∗*^	Separate	11 months/good
6, Jiang and Cheng, 1987 [6]	36, M	Visual disturbance	PA	Nonfunctioning	Transsphenoidal	Craniotomy	Nonfunctioning	Adamantinomatous^*∗*^	Separate^*∗*^	n.d./n.d.
7, Cusimano et al., 1988 [7]	62, F	Personality change, visual disturbance	CP	PRL (34 ng/ml)	Craniotomy (after VP shunt)	Craniotomy	PRL producing (micro)	Adamantinomatous	Separate	14 months/dead due to pulmonary embolism
8, Yoshida et al., 2008 [8]	29, M	Atrial fibrillation	PA	TSH	Transsphenoidal	None	TSH producing	Adamantinomatous	Encase	n.d./n.d.
9, Karavitaki et al., 2008 [9]	50, M	Headache, insomnia, reduced libido	PA	LH, FSH	Transsphenoidal	None	LH/FSH producing	Adamantinomatous	Encase	4 years/good (no recurrence)
10, Sargis et al., 2009 [10]	59, M	Visual disturbance	PA	LH, FSH	Craniotomy	None	LH/FSH producing	Adamantinomatous	Encase	n.d./diabetes insipidus
11, Moshkin et al., 2009 [11]	12, M	Partial hypopituitarism	CP	Nonfunctioning	Craniotomy	None	Nonfunctioning	Adamantinomatous	Encase	n.d./n.d.
12, Gokden and Mrak, 2009 [12]	47, M	Visual disturbance, headache	PA	Nonfunctioning	Transsphenoidal	None	Nonfunctioning	Adamantinomatous	Encase	1 year/good (no recurrence)
13, Jin et al., 2013 [13]	47, F	Visual disturbance, headache	PA	PRL (111 ng/ml) ACTH (116 pg/ml)	Transsphenoidal	Craniotomy	Nonfunctioning	Adamantinomatous	Separate	3 months/good (no recurrence)
14, Finzi et al., 2014 [14]	75, F	Diplopia	PA	PRL (54 ng/ml)	Transsphenoidal	None	Nonfunctioning	Adamantinomatous	Encase	10 months/good (no recurrence)
15, present case	48, M	Memory disturbance	CP	Nonfunctioning	Craniotomy	Transsphenoidal	Nonfunctioning	Adamantinomatous	Separate	10 years/good (no recurrence)

CP, craniopharyngioma; PA, pituitary adenoma; GH, growth hormone; PRL, prolactin; TSH, thyroid-stimulating hormone; LH, luteinizing hormone; FSH, follicle-stimulating hormone; ACTH, adrenocorticotropic hormone; VP, ventriculoperitoneal; ^*∗*^author's surmise based on reference; n.d., not described.
